# Transjugular Intrahepatic Portosystemic Shunt for the Treatment of Portal Hypertension in Noncirrhotic Patients with Portal Cavernoma

**DOI:** 10.1155/2014/659726

**Published:** 2014-04-29

**Authors:** Xuefeng Luo, Ling Nie, Biao Zhou, Denghua Yao, Huaiyuan Ma, Mingshan Jiang, Hailong Zhang, Xiao Li

**Affiliations:** Center of Interventional Radiology, West China Hospital, Sichuan University, 37 Guoxue Lane, Chengdu, Sichuan 610041, China

## Abstract

*Background*. The purpose of this study was to evaluate the safety and efficacy of transjugular intrahepatic portosystemic shunt (TIPS) placement in the management of portal hypertension in noncirrhotic patients with portal cavernoma. *Methods*. We conducted a single institution retrospective analysis of 15 noncirrhotic patients with portal cavernoma treated with TIPS placement. 15 patients (4 women and 11 men) were evaluated via the technical success of TIPS placement, procedural complications, and follow-up shunt patency. *Results*. TIPS placement was technically successful in 11 out of 15 patients (73.3%). Procedure-related complications were limited to a single instance of hepatic encephalopathy in one patient. In patients with successful shunt placement, the portal pressure gradient decreased from 25.8 ± 5.7 to 9.5 ± 4.2 mmHg (*P* < 0.001). TIPS dysfunction occurred in two patients during a median follow-up time of 45.2 months. Revision was not performed in one patient due to inadequate inflow. The other patient died of massive gastrointestinal bleeding in a local hospital. The remaining nine patients maintained functioning shunts through their last evaluation. *Conclusions*. TIPS is a safe and effective therapeutic treatment for noncirrhotic patients with symptomatic portal hypertension secondary to portal cavernoma.

## 1. Introduction

Chronic portal vein occlusion and cavernous transformation are a common cause of portal hypertension [1]. Despite the frequent absence of hepatocellular disease with preserved liver function, complete occlusion of the main portal vein or a dominant segment results in serious portal hypertensive complications including variceal bleeding, ascites, and portal cholangiopathy [1, 2]. In noncirrhotic patients with acute portal vein thrombosis, anticoagulation therapy with low molecular weight heparin followed by warfarin should be started immediately [1].

However, there is no established consensus guideline for the application of anticoagulation therapy in patients with chronic portal vein occlusion and portal cavernoma [1, 3]. For management of noncirrhotic portal hypertension due to portal cavernoma, there is no sufficient data on which of the several therapeutic options is preferred [1, 4, 5]. Endoscopic sclerotherapy and band ligation in noncirrhotic patients are as effective as in cirrhotic patients for control of acute variceal hemorrhage or prevention of recurrent variceal bleeding [6–8]. TIPS is considered as a rescue therapy, when technically applicable, as is routinely performed for cirrhotic patients. TIPS not only decreases the portal systemic pressure gradient but also prevents the extension of thrombosis into the superior mesenteric or splenic veins. However, the placement of TIPS in patients with portal cavernoma is regarded as more technically challenging and frequently associated with increased intraprocedural complications.

## 2. Methods

### 2.1. Patients

Between June 2005 and December 2011, we performed a TIPS procedure in 15 noncirrhotic patients (11 men and 4 women) who had documented variceal bleeding secondary to chronic portal vein thrombosis and associated cavernous transformation. Liver cirrhosis and hepatic cancer were ruled out on the basis of the history of liver disease, imaging studies, and the results of liver function tests. Liver biopsy was carried out if the diagnosis was in doubt. Portal cavernoma was defined as the presence of numerous venous collaterals at the hepatic hilum in the presence of portal vein thrombosis.

### 2.2. TIPS Procedure

The TIPS placement was performed by two experienced interventional radiologists using standard technique. Contrast-enhanced CT was utilized to locate the position of intrahepatic portal vein. Puncturing the portal vein exclusively via a transjugular approach with fluoroscopic guidance was intended primarily in all patients. If blind punctures failed, superior mesenteric arteriography was performed to localize the patent intrahepatic portal vein. When the portal vein was accessed, a hydrophilic guidewire was used to navigate peripheral to the cavernoma eventually accessing the patent portion of the main portal vein (MPV), splenic vein (SV), or superior mesenteric vein (SMV). Next, a portal venogram was performed and the portosystemic pressure gradient (PPG) was measured. The identified portal, splenic, or superior mesenteric vein thrombosis and the intrahepatic tract were dilated using a balloon angioplasty (PowerFlex, Cordis, Roden, Netherlands). ePTFE covered stents (Fluency, Bard, Murray Hill, USA) and/or bare stents (Smart, Cordis, Roden, Netherlands) were placed with their proximal end at the hepatocaval junction and their distal end in a patent portion of the portal venous system. Additional stents were placed to ensure complete coverage of stenosis or thrombosed segments. Finally, the PPG was remeasured and repeat portography was performed. Additional coil embolization of residual large variceal collaterals was performed if observed on the final portogram.

Intravenous heparin (4000 U) was administered to all the patients once the portal vein was entered. Low-molecular-weight heparin (LMWH) and warfarin were prescribed to all patients who had a successful TIPS creation. LMWH was continued for 3–5 days until target international normalized ratio (2-3) was achieved.

### 2.3. Follow-Up

The patients who were treated with TIPS were monitored for shunt patency by the same medical team in the gastroenterology clinic. In detail, duplex sonography was performed before discharge and at 1, 3, and 6 months after procedure and every 6 months thereafter or whenever clinically necessary.

### 2.4. Statistical Analysis

Results were expressed as means ± standard deviation. The PSG before and after TIPS placement was analyzed by paired Student's* t*-test. A *P* value of less than 0.05 was considered to indicate statistical significance. All calculations were performed by using SPSS version 20.0 software for Windows.

## 3. Results

### 3.1. Clinical Data

From June 2005 to December 2011, 15 noncirrhotic patients (11 males and 4 females) with a mean age of 29.1 ± 8.0 years (range 19–45 years) were treated with TIPS placement due to portal hypertension related variceal hemorrhage in our hospital. On clinical evaluation, active gastrointestinal bleeding was present in four patients on admission, and the other eleven patients had a history of variceal bleeding. 11 patients underwent a coagulopathy workup, of which nine patients had single or multiple thrombotic risk factors. Antithrombin III deficiency present in four patients, secondary thrombocytosis in three patients, protein C deficiency in three patients, protein S deficiency in one patient, and Factor V Leiden mutation in one patient were identified.

On contrast-enhanced CT scans, 11 patients had a completely occluded MPV, and four patients had segmental portal occlusion. The SMV was involved in nine patients and SV was involved in three out of six patients who had a spleen. A patient with Budd-Chiari syndrome (BCS) (case 5) had occlusion of two hepatic veins, as well as a long segment thrombosis of the inferior vena cava and deep veins of both lower extremities. The results of liver function tests showed normal liver function. Positive hepatitis B surface antigen was detected in four patients, but cirrhosis was ruled out by liver biopsy. Nine patients had undergone a prior splenectomy. One patient (case 7) had extensive portal vein thrombosis and cavernous formation fourteen months after orthotopic liver transplantation.

Patient characteristics, underlying prothrombotic disorders, imaging studies, laboratory results, and previous history of splenectomy are summarized in Table 1.

### 3.2. Procedure

The indications for TIPS placement were control of acute variceal bleeding in four patients refractory to conventional therapy and prevention of recurrent variceal bleeding in eleven patients with prior variceal hemorrhage who had failed endoscopic and/or beta-blocker therapy.

**Figure 1:**
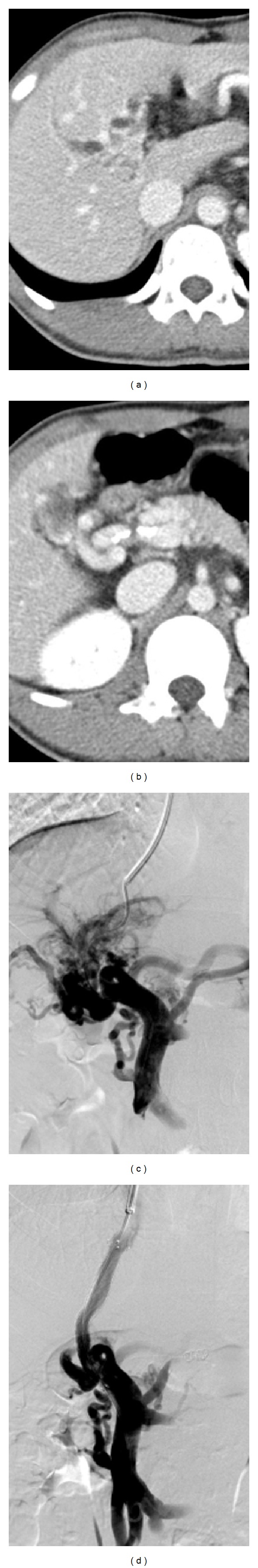
TIPS placement and recanalization of a segmental occluded main portal vein in a 26-year-old man with portal cavernoma. (a-b) CT scan shows occlusion of the portal vein at the hepatic hilum and partial patent main portal vein. (c) Portal venogram following access into the portal venous system reveals occlusion of the main portal vein and numerous collateral veins around the hilum. (d) Final portography obtained after TIPS placement with the distal end of the stent into the main portal vein demonstrates a good backflow through the shunt.

**Figure 2:**
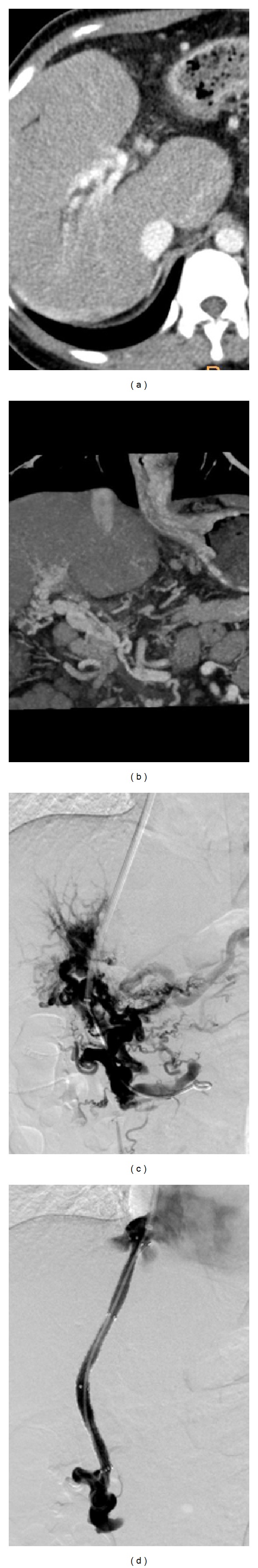
TIPS placement in a 21-year-old woman with portal cavernoma. (a-b) CT scan shows the obliteration of the original main portal vein and superior mesenteric vein, as well as the periportal collateral veins supplying the intrahepatic veins. (c) Direct portal venogram after catheterization into the portal vein reveals only collateral vessels and varices. (d) Final portal venogram obtained after TIPS placement demonstrates a good decompression of the portal venous system.

TIPS placement was technically successful in 11 (73.3%) out of 15 patients. In cases 3, 12, and 14, although the intrahepatic portal vein was accessed, the guidewire could not be negotiated across the occluded MPV despite numerous attempts. In case 1, no patent intrahepatic portal vein was visualized during the procedure. In eight patients, single covered stent was placed. In other three patients, additional stents (bare stents in one patient and covered stents in two patients) were placed in a coaxial fashion to ensure sufficient shunt flow. 10 mm diameter stents were deployed in 9 patients (Figure 1); 8 mm diameter stents were deployed in 2 patients in a large collateral vein since recanalization of the MPV failed (Figure 2). The average PPG decreased from 25.8 ± 5.7 to 9.5 ± 4.2 mmHg following the procedure (*P* < 0.001). In two patients, coil embolization of residual large collateral varices was performed to increase shunt flow. All TIPS creation was performed via transjugular approach only; neither transhepatic nor transsplenic approaches were utilized in our series.

Hemostasis was achieved in two patients with prior active variceal bleeding immediately after successful TIPS placement. One patient developed hepatic encephalopathy (West Haven criteria grade 3) within two days after TIPS placement and was successfully treated with oral rifaximin and rectal enemas. No other major procedure-related complications such as intra-abdominal hemorrhage or clinically evident pulmonary embolization were observed.

### 3.3. Follow-Up

The median follow-up time was 45.2 months. Two patients (cases 5 and 10) experienced 3 episodes of hepatic encephalopathy and were successfully treated with conventional medical treatment. TIPS dysfunction occurred in two patients (cases 9 and 11) 4 and 10 months after the procedure, respectively. Revision was not performed in the first patient (case 9) who had a completely occluded MPV and SMV. We decided not to insert an additional stent graft as it was impossible to obtain adequate inflow to keep the shunt patent. This patient received beta-blocker therapy in combination with EVL; however, two episodes of gastrointestinal bleeding still occurred during the follow-up. The other patient (case 11) presented with massive gastrointestinal bleeding and was sent to a local hospital. Unfortunately, this patient succumbed to blood loss, possibly due to the delayed emergency treatment. The other nine patients remained alive and no shunt insufficiency was observed throughout the follow-up period.

One patient (case 3), in whom placement of TIPS failed, died of massive upper gastrointestinal bleeding five days after the attempted procedure. One patient (case 14) died of sepsis and multiorgan system failure after 15 months, for reasons unrelated to TIPS. Another two patients (cases 1 and 12) remained alive until the last clinical evaluation, although a total of four episodes of variceal rebleeding occurred. Technical details and follow-up results were summarized in Table 2.

## 4. Discussion

Noncirrhotic portal hypertension due to chronic portal vein thrombosis and portal cavernoma formation is relatively rare and almost always associated with underlying prothrombotic disorders [1]. Chronic portal vein thrombosis usually results in the cavernous transformation of the portal vein, which manifests numerous porto-portal collateral veins at the liver hilum. The diagnosis of portal cavernoma is not difficult to establish on the basis of ultrasound, CT, or MRI. The most common clinical presentation on admission is gastrointestinal bleeding due to ruptured gastroesophageal varices, as well as hypersplenism, ascites, and/or portal cholangiopathy. Liver function tests are usually normal in noncirrhotic patients with portal cavernoma in the absence of underlying liver disease. The prognosis of noncirrhotic patients with portal cavernoma is good. Mortality is mainly related to concomitant disorders leading to extrahepatic portal vein thrombosis and not to complications of portal hypertension [9, 10].

Previous clinical studies have shown that the technical success rate of TIPS in patients with cavernoma can be significantly variable [2, 11–14]. The recent published series from Fanelli et al. (*n* = 13, mean follow-up 17.4 months) and Qi et al. (*n* = 20, median follow-up 19.9 months) showed TIPS success rate of 83% and 35%, respectively [2, 14]. Variety between different clinical observations is possibly due to the severity and extension of thrombosis, the history of portal cavernoma, and whether or not a larger collateral vein existed among patients who were included. Compared with previous reports, our cohort has a comparable technical success rate (73%, 11/15). Based on our experience, we presumed that two key factors may determine the success of TIPS placement in these patients.

Firstly, the severity and extension of portal vein thrombosis should be carefully evaluated before the procedure. Partial and central thrombosis of the MPV or SMV significantly increase the change of TIPS failure. Once the guidewire is negotiated across the thrombosed segment reaching the patent part of the MPV or SMV, TIPS placement is readily performed following portal vein angioplasty. Conversely, if thrombosis extends deep into distal branches of the SMV, even if the guidewire can be advanced beyond the thrombus, stents cannot be placed as there is insufficient inflow to maintain shunt patency.

Secondly, the existence of a large porto-porto collateral vein which communicates with the varices is another rational reason to place the stent. Noncirrhotic patients with chronic portal vein occlusion and cavernous transformation usually present with multiple tortuous and small collateral vessels at the liver hilum on preoperative imaging studies. TIPS placement without sufficient inflow may cause early shunt dysfunction and cannot obtain a good decompression of the portal venous system. In our series, all successful cases had either a patent SMV or a large porto-porto collateral vein. In our view, patients without aforementioned basic conditions should be considered technically contradicted in TIPS placement.

It should be noted that a technical success rate of 73% in our study was achieved via the transjugular approach alone. Neither transhepatic nor transsplenic approaches were used for catheterization in the portal venous system. The transhepatic approach has been widely adopted by most interventional radiologists for various portal vein interventions including portal vein stent placement and portal vein embolization [15, 16]. The traditional TIPS procedure could be carried out after the portal vein catheterization and angioplasty. Our unit has relatively large blind transjugular intrahepatic puncture experience (more than 1000 TIPS procedures since 2005). Wedged CO_2_ indirect portography showed circuitous collateral vessels with poor identification of intrahepatic portal branches. Catheterization of the portal venous system could also be achieved by calculating the needle throw direction and length by triangulating with CT images. In general with cases of cavernoma, the challenge is not portal access but the occluded cavernous segment and reaching the patent part of the MPV or SMV. Unlike general cirrhotic patients with portal vein thrombosis, the original MPV in noncirrhotic patients with portal cavernoma is usually obliterated and replaced by a fibrotic cord. Balloon angioplasty of the tortuous and small collateral vessels has a high risk of rupture and life-threatening intra-abdominal hemorrhage.

Although transhepatic portal vein angioplasty is of great use to recanalize the true lumen of the occluded portal vein, the application of this route should be cautiously considered in patients with portal cavernoma. However, transhepatic portal vein angioplasty may still be performed if the catheterization was initiated from a normal intrahepatic portal vein into the MPV. Transsplenic approach has been well described as a valuable method for portal venous intervention [17–19]. This method provides a direct and straight line of access to the portal vein circulation as well as favorable force vectors for handling wires and catheters, especially in the presence of intrahepatic portal vein occlusion or ascites. The principle possible complication of this approach is intra-abdominal hemorrhage from the splenic puncture site and intrasplenic hematomas in the presence of portal hypertension, splenomegaly, and thrombocytopenia [17]. Another drawback to the transsplenic approach may be difficulty in catheter advancement within the tortuous course of the splenic vein. In patients with portal vein thrombosis, the usefulness of this route would be impaired if the thrombosis extended deep into the SV. We did not try the transsplenic approach in the present study, as three patients underwent prior splenectomy and devascularization, and the other patient showed complete SV occlusion with collateral vessels around the splenic hilum.

Incidentally, the high incidence of patients having undergone prior splenectomy in the present study is potentially related to the older practice of inappropriate yet widely accepted use of surgical therapy in the treatment of portal hypertension in our country, especially in pediatric patients.

Whether or not long-term TIPS patency is affected by the patient's underlying prothrombotic disorder is not yet clear. Some authors assumed that TIPS dysfunction rate may be increased in these patients due to a persistent untreated prothrombotic state [2, 14]. In a recent clinical study which showed a similar shunt patency between cirrhotic patients with and without extrahepatic portal vein obstruction, heparin was prescribed in all the patients for 10 days and was prolonged to 20 days in patients with PVT without thrombophilia [11]. In the present study, all 11 patients who underwent TIPS successfully received lifetime oral anticoagulation therapy and TIPS dysfunction occurred in two of 11 patients (18%) during a median follow-up time of 45.2 months. Considering the small number of patients included, the incidence of shunt dysfunction herein lacks predictive power.

From our point of view, anticoagulation will contribute to complete recanalization of the portal venous system and prevent recurrent thrombosis. In the meantime, we recommend the use of ePTFE covered stent to maximize long-term TIPS as demonstrated by preliminary studies in cirrhotic patients [20].

The primary limitation of our work is its small size, retrospective nature, and lack of patient randomization. Unfortunately, noncirrhotic portal hypertension due to chronic portal vein occlusion is uncommon and will be difficult to valuate with prospective studies. Also, the usefulness of transhepatic approach in these patients is unclear in this study.

Despite the small sample size, we conclude that TIPS placement is a safe and effective treatment for relieving cavernoma associated portal hypertensive variceal hemorrhage in noncirrhotic patients. While TIPS in this patients population is technically more challenging, it is not contradicted and remains a viable option for symptomatic portal hypertension within experienced institutions.

## Figures and Tables

**Table 1:** Patient characteristics, previous history of splenectomy, imaging studies, and laboratory results.

Patient number/age (y)/sex	Underlying prothrombotic disorders	Imaging studies	TBIL (*μ*mol/L)	ALT (U/L)	AST (U/L)	ALP (U/L)	WBC (109/L)	Platelet (109/L)	Splenectomy
1/35/F	N/A	Total occluded MPV and SMV	10.2	12	15	79	4.94	122	Yes

2/24/M	N/A	Partial occluded MPV	9.4	23	50	73	2.76	45	Yes

3/39/M	N/A	Total occluded MPV and SV and partial occluded SMV	11.3	11	25	75	9.77	6	No

4/19/M	Antithrombin III deficiency, thrombocytosis	Total occluded MPV	15.3	57	86	59	1.83	472	Yes

5/25/F	Protein C deficiency, antithrombin III deficiency	Total occluded MPV and partial occluded SMV and SV	36.4	37	32	166	4.37	25	No

6/26/M	N/A	Partial occluded MPV	10.4	17	25	112	2.51	71	Yes

7/25/F	Thrombocytosis	Total occluded MPV	6.4	29	33	70	2.63	653	Yes

8/40/M	Antithrombin III deficiency	Partial occluded MPV	7.7	19	26	96	4.17	216	No

9/21/F	No	Total occluded MPV and SMV	15.6	29	19	101	14.26	185	Yes

10/19/M	Antithrombin III deficiency	Total occluded MPV and partial occluded SMV	19.6	16	33	68	3.41	51	No

11/34/M	Protein C deficiency	Total occluded MPV, partial occluded SMV and SV	13.8	13	26	72	2.42	52	No

12/45/M	No	Total occluded MPV and SMV	19	13	21	145	2.15	277	Yes

13/31/M	Thrombocytosis	Total occluded MPV and SMV	10.8	15	23	67	1.27	517	Yes

14/29/M	FVL mutation, protein C deficiency	Total occluded MPV and partial occluded SMV	6	26	24	63	3.54	189	Yes

15/24/M	Protein S deficiency	Partial occluded MPV	8.1	10	16	38	7.68	92	No

TBIL: total bilirubin, ALT: alanine aminotransferase, AST: aspartate aminotransferase, ALP: alkaline phosphatase, WBC: white blood cell, MPV: main portal vein, SMV: superior mesenteric vein, SV: splenic vein, and FVL mutation: Factor V Leiden mutation.

**Table 2:** Technical details and follow-up results.

Patient number	Technique success of TIPS	Stent brand	Stent placement	Hepatic encephalopathy	TIPS dysfunction	Survival (months after TIPS)
1	No	—	—	—	—	Alive
2	Yes	Fluency	10 × 60 mm	No	No	Alive
3	No	—	—	—	—	Dead (5 days)
4	Yes	Fluency	10 × 80 mm	No	No	Alive
5	Yes	Fluency, SMART	10 × 60 mm 10 × 60 mm	Yes	No	Alive
6	Yes	Fluency	10 × 80 mm	No	No	Alive
7	Yes	Fluency	10 × 80 mm	Yes	No	Alive
8	Yes	Fluency	10 × 80 mm	No	No	Alive
9	Yes	Fluency	8 × 80 mm × 2	No	Yes	Alive
10	Yes	Fluency	10 × 80 mm	Yes	No	Alive
11	Yes	Fluency	10 × 60 mm × 2	No	Yes	Dead (10 months)
12	No	—	—	—	—	Alive
13	Yes	Fluency	8 × 80 mm	No	No	Alive
14	No	—	—	—	—	Dead (15 months)
15	Yes	Fluency	10 × 60 mm	No	No	Alive
